# Management of patients with placenta accreta in association with fever following vaginal delivery

**DOI:** 10.1097/MD.0000000000006279

**Published:** 2017-03-10

**Authors:** Liuying Zhong, Dunjin Chen, Mei Zhong, Yutian He, Chunhong Su

**Affiliations:** a Department of Nanfang Hospital of Southern Medical University; b Department of The 3rd Affiliated Hospital of Guangzhou Medical University, Obstetric Critical Care Center of Guangzhou, Key Laboratory for Major Obstetric Disease of Guangzhou Province, Guangzhou, China.

**Keywords:** placenta accreta, conservative management, hysterectomy, fever

## Abstract

This study aims to analyze the clinical characteristics and to manage patients with retained placenta left in situ accompanied by fever following vaginal delivery.

Twenty-one patients with retained placenta in association with fever following vaginal delivery were enrolled and managed at the maternity department of our university hospital between 2012 and 2014.

All patients had risk factors for development of placenta accreta: previous cesarean sections (4/21), previous curettage (15/21), or uterine malformations (7/21). Placenta accreta was diagnosed following vaginal delivery in all patients, and manual removal of the placenta was attempted in 20 of 21 patients. The placenta left in situ was partial in 19 patients and was complete in 2 patients. All patients were managed with a multidisciplinary approach. Mifepristone was administrated to 16 patients. Fourteen patients received uterine artery embolization. Eleven patients were treated with ultrasound-guided curettage within 24 hours following delivery. Seven patients needed delayed-hysterectomy due to development of complications.

Intrauterine operations during labor are not recommended if placenta accreta occurs in the fundus and/or in the cornual region of the uterus. Antibiotic treatment, interventional therapy, and ultrasound-guided curettage within 24 hours following vaginal delivery are the recommended conservative management strategies.

## 1 Introduction

Placenta accreta is an obstetrical complication where the placenta becomes firmly adherent to the uterine wall.^[1]^ Placenta accreta can lead to considerable maternal morbidity and mortality due to hemorrhage, infection, or other surgical complications such as those resulting from hysterectomy.^[1,2]^ Retained placenta accreta is usually a rare condition, but its prevalence is increasing due to the rise in the rate of deliveries by Cesarean section.^[1–4]^

A multidisciplinary integrated management strategy at an appropriate tertiary care center is essential, in order to reduce the mortality and morbidity associated with placenta accrete.^[5,6]^ The optimum management strategies include either conservative management of the placenta left in situ, or surgical management which mainly includes hysterectomy.^[6]^ The treatment modalities of conservative approach include use of methotrexate, uterine artery embolization, dilation and curettage, and hysteroscopic loop resection.^[2,6,7]^ The decision for choosing conservative management or hysterectomy depends on, the extent of placental infiltration, patient's hemodynamic and infection status, and her desire for retaining fertile.

Fever is a frequent symptom occurring in cases of complicated placenta accreta and is an indication of infection. This condition is associated with significant maternal morbidity and mortality. Infection of the placenta left in situ may progress to infectious toxic shock and hemorrhage, and therefore, requires special attention and close monitoring. However, fever during postpartum period may not always indicate infection, as in some patients it may represent tissue necrosis.^[8]^

The purpose of this retrospective study is to analyze the clinical characteristics, management strategies, and outcomes in 21 patients with placenta accreta and having concomitant fever in the peripartum period. The author's experiences with various management strategies employed to reduce maternal morbidity are evaluated and discussed.

## 2 Materials and methods

This a retrospective study conducted at the 3rd Affiliated Hospital of Guangzhou Medical University, Obstetric Critical Care Center of Guangzhou. The study evaluated the 21 patients who had developed placenta accreta and suffered with concomitant fever for various clinical characteristics and management strategies, between January 2012 and August 2014. This study was approved by the ethical committee of the 3rd Affiliated Hospital of Guangzhou Medical University, China. Clinical information was gathered from the written delivery reports, and databases of the Pathology and Radiology Departments. The data collected included: patient medical, obstetric, and gynecologic history; time of diagnosis, intrapartum and postpartum management, maternal and neonatal complications.

Initial suspicion of placenta accreta was based on ultrasound result, or difficulty encountered in manual removal of the placenta. The patients were finally concluded to have placenta accreta by magnetic resonance imaging (MRI), histopathological examination of surgical placental, or hysterectomy specimens. The criteria for defining intra-abdominal infection was persistent fever >38.0°C, leukocytosis, and abdominal pain.^[8]^ Endomyometritis is defined as sepsis in the inner lining of the uterus.

## 3 Results

### 3.1 Patients’ clinical data

The information regarding the clinical and delivery data of the patients is presented in Table 1. The mean age of the 21 women enrolled in our study was 28 years (range 23–33 years). Average gravidity and parity was 2.90 (range 1–6) and 1.19 (range 0–3), respectively. All patients had identifiable risk factors for development of abnormal placental implantation including previous cesarean section (4/21), previous curettage (range 0–3 times, average 1 time; 15/21 patients), and uterine malformations (7/21). Of the 7 women who had uterine malformations, 2 had septate uterus, 2 had uterus didelphys, 2 had bicornuate uterus, and 1 had arcuate uterus. There was premature rupture of membranes in 9 women, and the rupture to labor time was between 3 hours and 9 days.

**Table 1:**
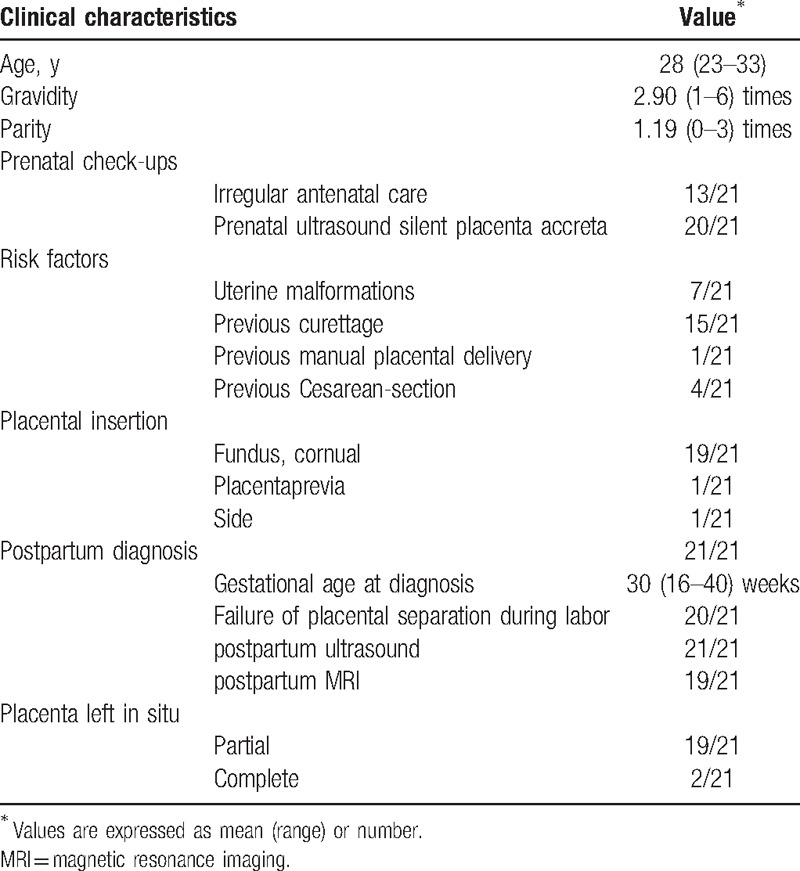
Demographic and clinical characteristics of patients with placenta accreta.

### 3.2 Diagnosis of placenta accreta

Placenta accreta can be identified during antenatal visits in high-risk pregnancies and also be detected by ultrasound. Unfortunately, in our study, even though at least 1 antenatal ultrasound was performed in 20 women, none were suspected to be having placenta accreta. The placenta accreta was identified in 20 patients at the time of delivery when attempts to remove placenta manually failed. Clinical diagnosis of placenta accreta was subsequently confirmed by postpartum ultrasound and MRI. Placenta accreta was diagnosed in 8 patients before 28 weeks of gestational age, the earliest gestational age being 16 weeks. Placental insertion in these 8 patients was mostly at the fundus and/or at the cornual region of the uterus (90.48%; Table 1).

### 3.3 Fever accompanying placenta accreta, and investigations for etiology

All patients had persistent fever which lasted from 2 days to 3 months. Fever had occurred before the termination of pregnancy in 3 patients, of whom 1 had an upper respiratory tract infection, and the other 2 patients had vaginal bleeding. The occurrence of fever in the remaining 18 patients was after manual removal of placenta and/or after curettage, except for 1 patient who had preterm premature rupture of membranes. The range of temperature was between 37.6°C and 42°C. Fever was remittent in nature in 19 patients, with the highest temperature of more than 39°C, which accompanied vaginal bleeding or abdominal pain. Temperature had increased markedly the day after use of methotrexate or curettage, in 6 of the patients.

Patients’ white blood cell (WBC), neutrophils (N), C-reactive protein (CRP), and procalcitonin (PCT) were monitored upon admission, prenatally, up to discharge (Table 2). The normal range of these parameters are as follows: WBC, (4–8) × 10^9^/L; N, 50–70%; CRP, 0–5 mg/L and PCT < 0.05 ng/mL. Four patients who underwent delayed-hysterectomies due to septic shock had abnormally high WBC (>20 × 10^9^/L) at least once during investigations. The remaining patients had increasingly reactive WBC and neutrophils in the postpartum period. Their WBC levels had decreased after placental separation. The samples examined for identification of etiology were blood, urine, vaginal secretions, and bone marrow culture. Blood, urine, and vaginal samples were collected 24 hours after invasive placenta separation. Second samples were collected a week after the first sample, and the last sample was collected prior to discharge. Blood was drawn for culture from patients when the fever had reached 38.0°C or above, or 1 week following antibiotics administration in patients who had persistent remittent fever. Blood culture was negative in 6/21 cases, whereas at least 1 pathogen was detected in the remaining 15 patients. Nine patients had *Escherichia coli*, 6 had *Enterococcus faecalis*, 3 had candida, 2 had bird enterococci plus Gram positive cocci, and 1 had *Staphylococcus aureus* and *epidermidis*. Bone marrow examination of 1 patient revealed presence of bone marrow hyperplasia, and there were cytoplasmic toxic particles and vacuoles in granulocytes.

**Table 2:**
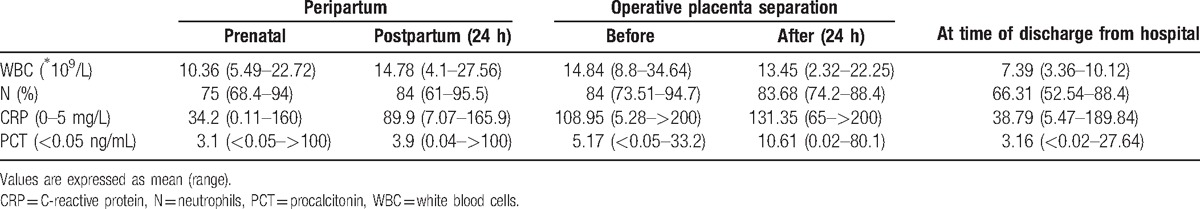
The infection index in patients with retained placenta accreta with fever.

### 3.4 Peripartum and postpartum management

Management during the peripartum and postpartum periods is summarized in Table 3. All women had a vaginal delivery. The presenting part during delivery was head in 10/21 (47.61%) patients, breech in 3/21 (14.29%) patients, the remaining 8/21 (38.09%) patients had abortions, and that 2 of them had taken abortion pills due to fetal malformations and 2 patients had stillborns. Manual placenta removal was attempted in all patients but had failed in 20/21 patients (95.24%). One patient was suspected to have placenta accreta during delivery, and thus, no attempt was made to remove placenta manually.

**Table 3:**
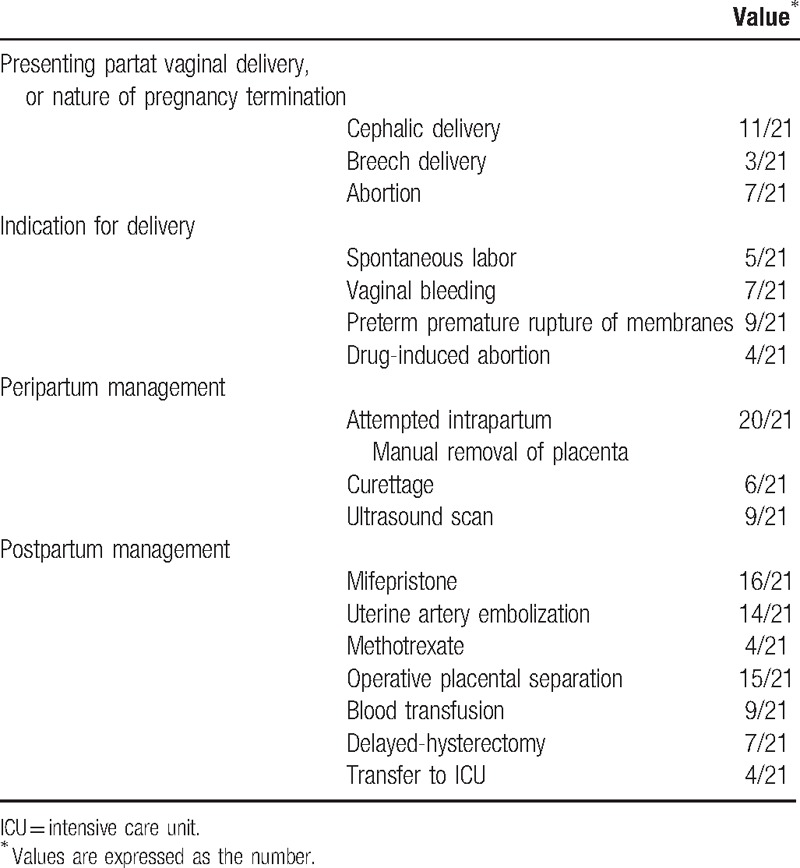
Management of patients with retained placenta accreta with fever.

Postpartum management of retained placenta accreta includes treatment with methotrexate and mifepristone, uterine artery embolization, dilation and curettage, and delayed hysterectomy. Initial symptom-based antibiotic treatment includes cephalosporins used in combination with metronidazole. The broad-spectrum antibiotics were used as a final resort to manage aggravating symptoms and signs of infection, and based on the laboratory report.

Fourteen (66.67%) patients underwent uterine artery embolization. Of them, 4 patients had received methotrexate (100 mg) during the procedure, 11 patients needed curettage under B-mode ultrasound guidance within 24 hours, and the remaining 3 were subjected to hysterectomy. Among the 11 patients who underwent curettage, an average of 1 to 2 curettages were performed and the associated blood loss was in the range of 5 to 50 mL. Overall, 7 patients needed hysterectomies during the first 48 days (mean 18.83 days) following vaginal delivery. The indication for hysterectomy included intrauterine infections (2 cases), pelvic abscess (1 case), and complicated septic shock (4 cases). Among these 7 patients, 6 patients were parous. The remaining one was 25-year-old primigravida who had spontaneous abortion associated with fever that originated from upper respiratory tract infection. Attempt to remove the placenta resulted in series of complications such as acidosis, toxic myocarditis, coagulation disorders, and multiple organ failure (MOF). This patient was taken for hysterectomy 1 day after abortion. One of the patients needed emergency hysterectomy due to uterine perforation during the second curettage. The cause of the emergency hysterectomy was suspected to be placenta percreta to the bladder with evidence of infection.

### 3.5 Maternal and neonatal outcome

There was no maternal death among the 21 patients. Nine patients had received blood transfusion: red blood cell range 2–10 units, plasma 0–800 mL during labor, and 2 patients had suffered hemorrhagic shock. Eighteen patients had suffered from anemia (hemoglob in range 53–94 g/L), 7 had hypoproteinemia (range 20–29.3 g/L), 10 had endomyometritis, 5 had septic shock, 2 had intra-abdominal infection, and 1 had MOF. Four patients were admitted to the intensive care unit (ICU) (average stay 3.75 days, range 1–6 days) and required postoperative mechanical ventilation and dialysis.

There were 24 fetuses (with 2 pair of twins), 11 neonates were alive, 5 neonates were stillborn, 6 fetuses were aborted, and there were 2 neonatal deaths. The mean gestational age at delivery of live born neonates was 37.3 (range 29–40) weeks. The mean weight of liveborn neonates was 2726.36 g (range 1050–3800 g). Two neonates were admitted in the neonatal intensive care unit (NICU). One neonate was admitted because of premature delivery (29 weeks) and associated low birth weight (1050 g). The other was because of premature delivery (34 weeks) with mild asphyxia.

## 4 Discussion

The incidence of placenta accrete is rising due to the increased frequency of Cesarean section.^[1]^ Placenta accreta can be associated with serious bleeding in late pregnancy and in labor.^[1]^ Hysterectomy after diagnosis of placenta accreta can lead to considerable maternal morbidity and mortality due to hemorrhage, infection, or other surgical complications.^[1–2]^ In our study, none of the patients were diagnosed to have placenta accreta antenatally. This could be due to 1 or more of the following reasons: irregular prenatal visits, placental implantation at the fundus and/or at the cornua, and failure of the obstetrician to provide valuable data to the sonologist. Because of the unawareness of the placenta accreta, manual removal was attempted in 20 of the patients, but the attempts had failed. A multidisciplinary conservative approach was adopted to manage all these patients in order to retain fertility, and primarily focused on reducing and controlling fever. Nineteen patients had partial placenta left in situ, whereas 2 patients had complete placenta left in situ. Conservative management was successful in 15 patients, and uteruses were preserved in these patients. Our study supports the possibility of conservative management for patients who are diagnosed to have placenta accreta/increta in the postpartum period.^[9]^ However, despite our efforts towards conservative management, 7 patients needed delayed hysterectomy.

We recommend utilization of imaging modalities such as ultrasound or MRI for the diagnosis and follow-up of women with abnormally invasive placentation.^[10–13]^ Three known risk factors for placenta accrete are previous cesarean delivery, placenta previa, and anterior placentation.^[14]^ Patients with these risk factors should be closely monitored with sonography.^[10–13]^ Ultrasound has served traditionally as the initial diagnostic modality, but the use of MRI has increased steadily in practice due to improved accuracy.^[13]^ We recommend using MRI in patients with high risk factors in case ultrasound fails to detect abnormal placenta plantation antenatally. However, ultrasound can be used in the postpartum period to assess the size of the placenta left in situ, its relationship to the uterus, and blood flow.^[11,12]^

Fever is a frequently reported complication of placenta accreta. In most of the cases, fever is secondary to endomyometritis or florid sepsis. However, fever can also be due to tissue necrosis without any inflammatory response.^[5]^ In our study, 2 among the 6 patients with negative etiologies and placental pathologies after hysterectomy, had endomyometritis. The remaining 4 patients had placental tissue necrosis which was confirmed by the pathological examination. There was infection in the remaining 15 patients; which included sepsis in 11 patients. Five of these 11 patients had septic shock. Microbiology cultures were positive in 17/21 patients from either blood, urine, or vaginal fluid culture, and *E coli* was the common pathogen identified.

If the placental implantation abnormality is suspected, then such patients should be referred to an experienced medical team at a tertiary care center to confirm the diagnosis.^[15]^ Based on our experiences, we suggest avoiding forced manual placenta removal if the placenta has infiltrated the perimetrium and surrounding tissues. Ultrasound and/or MRI should be performed to confirm placenta implantation, and placenta removal should be conducted by experienced obstetrician under ultrasound guidance. Intrauterine procedures during labor are not recommended if placenta accreta occurs in the fundus and/or in the cornua of the uterus. Preparation for anti-infective treatment or hysterectomy should be made.

We performed curettage in 11 patients within 24 hours after delivery. Our study has shown that curettage is an effective conservative management strategy. It reduces hemorrhage and also helps to preserve the uterus and fertility. We recommend performing curettage under ultrasound guidance so as to avoid inversion or perforation of the uterus. Patients with serious complications such as septic shock need delayed hysterectomy, which can be performed 4 to 6 weeks after cesarean delivery. This lag time allows the periuterine vascularization to recede and to prevent late onset massive hemorrhage or severe infection.^[13]^ Within our cohort, the mean lag time was 16.1 days (range 1–48 days).

In conclusion, 21 patients were diagnosed to have placenta accreta following vaginal delivery at our hospital between 2012 and 2014. A conservative and multidisciplinary approach helped to preserve uterus in 14 patients, whereas 7 patients needed hysterectomy due to development of infections and other complications. We hope that our experience and management strategy provides valuable guidance for the treatment of placenta accreta, diagnosed in the peripartum period.

## References

[1] Wortman AC Alexander JM Placenta accreta, increta, and percreta. Obstet Gynecol Clin North Am 2013;40:137–54. 23466142 10.1016/j.ogc.2012.12.002

[2] Oppenheimer L Diagnosis and management of placenta previa. J Obstet Gynaecol Canada 2007;29:261–73. 10.1016/S1701-2163(16)32401-X 17346497

[3] Pather S Strockyj S Richards A Maternal outcome after conservative management of placenta percreta at caesarean section: a report of three cases and a review of the literature. Aust N Z J Obstet Gynaecol 2014;54:84–7. 24471850 10.1111/ajo.12149

[4] Perez-Delboy A Wright JD Surgical management of placenta accreta: to leave or remove the placenta? Bjog 2014;121:163–9. discussion 169–170. 24373590 10.1111/1471-0528.12524

[5] Yu M Liu XY Dai Q Diagnosis and treatment of placenta accreta in the second trimester of pregnancy. Zhongguo Yi Xue Ke Xue Yuan Xue Bao 2010;32:501–4. 21050552 10.3881/j.issn.1000-503X.2010.05.006

[6] Eller AG Porter TF Soisson P Optimal management strategies for placenta accreta. Bjog 2009;116:648–54. 19191778 10.1111/j.1471-0528.2008.02037.x

[7] Arulkumaran S Ng CS Ingemarsson I Medical treatment of placenta accreta with methotrexate. Acta Obstet Gynecol Scand 1986;65:285–6. 3739639 10.3109/00016348609155187

[8] Henrich W Fuchs I Ehrenstein T Antenatal diagnosis of placenta percreta with planned in situ retention and methotrexate therapy in a woman infected with HIV. Ultrasound Obstet Gynecol 2002;20:90–3. 12100427 10.1046/j.1469-0705.2002.00691.x

[9] Peiffer S Reinhard J Reitter A Conservative management of placenta accreta/increta after vaginal birth. Geburtshilfe Frauenheilkd 2012;72:940–4. 25308979 10.1055/s-0032-1327827 PMC4168407

[10] Tanaka YO Shigemitsu S Ichikawa Y Postpartum MR diagnosis of retained placenta accreta. Eur Radiol 2004;14:945–52. 15045519 10.1007/s00330-004-2266-8

[11] Warshak CR Eskander R Hull AD Accuracy of ultrasonography and magnetic resonance imaging in the diagnosis of placenta accreta. Obstet Gynecol 2006;108:573–81. 16946217 10.1097/01.AOG.0000233155.62906.6d

[12] Comstock CH Love JJ Jr Bronsteen RA Sonographic detection of placenta accreta in the second and third trimesters of pregnancy. Am J Obstet Gynecol 2004;190:1135–40. 15118654 10.1016/j.ajog.2003.11.024

[13] Levine D Hulka CA Ludmir J Placenta accreta: evaluation with color Doppler US, power Doppler US, and MR imaging. Radiology 1997;205:773–6. 9393534 10.1148/radiology.205.3.9393534

[14] Timmermans S van Hof AC Duvekot JJ Conservative management of abnormally invasive placentation. Obstet Gynecol Surv 2007;62:529–39. 17634154 10.1097/01.ogx.0000271133.27011.05

[15] Chantraine F Nisolle M Petit P Individual decisions in placenta increta and percreta: a case series. J Perinat Med 2012;40:265–70. 22505505 10.1515/jpm-2011-0156

